# Trans-Cinnamaldehyde Alleviates Amyloid-Beta Pathogenesis via the SIRT1-PGC1α-PPARγ Pathway in 5XFAD Transgenic Mice

**DOI:** 10.3390/ijms21124492

**Published:** 2020-06-24

**Authors:** Jimin Do, Namkwon Kim, Seung Ho Jeon, Min Sung Gee, Yeon-Joo Ju, Jong-Ho Kim, Myung Sook Oh, Jong Kil Lee

**Affiliations:** 1 Department of Biomedical Science and Technology, Graduate School, Kyung Hee University, 26 Kyungheedae-ro, Dongdaemun-gu, Seoul 02447, Korea; djimin93@gmail.com; 2 Department of Life and Nanopharmaceutical Sciences, Graduate School, Kyung Hee University, 26 Kyungheedae-ro, Dongdaemun-gu, Seoul 02447, Korea; kop03@khu.ac.kr; 3 Department of Fundamental Pharmaceutical Science, Graduate School, Kyung Hee University, 26 Kyungheedae-ro, Dongdaemun-gu, Seoul 02447, Korea; bawoojang@khu.ac.kr (S.H.J.); 2017315113@khu.ac.kr (M.S.G.); yeonj00@khu.ac.kr (Y.-J.J.); 4 Department of Pharmaceutical Science, College of Pharmacy, Kyung Hee University, 26 Kyungheedae-ro, Dongdaemun-gu, Seoul 02447, Korea; jonghokim@khu.ac.kr; 5 Department of Oriental Pharmaceutical Science, College of Pharmacy, Kyung Hee University, 26 Kyungheedae-ro, Dongdaemun-gu, Seoul 02447, Korea

**Keywords:** Alzheimer’s disease, amyloid-β, β-secretase, trans-cinnamaldehyde, SIRT1, PGC1α, PPARγ

## Abstract

Abnormal amyloid-β (Aβ) accumulation is the most significant feature of Alzheimer’s disease (AD). Among the several secretases involved in the generation of Aβ, β-secretase (BACE1) is the first rate-limiting enzyme in Aβ production that can be utilized to prevent the development of Aβ-related pathologies. Cinnamon extract, used in traditional medicine, was shown to inhibit the aggregation of tau protein and Aβ aggregation. However, the effect of trans-cinnamaldehyde (TCA), the main component of cinnamon, on Aβ deposition is unknown. Five-month-old 5XFAD mice were treated with TCA for eight weeks. Seven-month-old 5XFAD mice were evaluated for cognitive and spatial memory function. Brain samples collected at the conclusion of the treatment were assessed by immunofluorescence and biochemical analyses. Additional in vivo experiments were conducted to elucidate the mechanisms underlying the effect of TCA in the role of Aβ deposition. TCA treatment led to improvements in cognitive impairment and reduced Aβ deposition in the brains of 5XFAD mice. Interestingly, the levels of BACE1 were decreased, whereas the mRNA and protein levels of three well-known regulators of BACE1, silent information regulator 1 (SIRT1), peroxisome proliferator-activated receptor γ (PPARγ) coactivator 1α (PGC1α), and PPARγ, were increased in TCA-treated 5XFAD mice. TCA led to an improvement in AD pathology by reducing BACE1 levels through the activation of the SIRT1-PGC1α-PPARγ pathway, suggesting that TCA might be a useful therapeutic approach in AD.

## 1. Introduction

Alzheimer’s disease (AD) is a chronic neurodegenerative disease and is the most common cause of dementia, accounting for 60% to 70% of senile dementia. The clinical symptoms of AD include memory impairment and cognitive decline, and the typical pathological features are deposition of amyloid-β (Aβ) plaques and synaptic loss [1]. Despite numerous efforts, the cause of AD has not yet been fully elucidated. At present, there are no therapeutic approaches that are effective in prevention or treatment of AD. Currently approved therapeutics for AD by the U.S. Food and Drug Administration are based on the cholinergic hypothesis; these drugs, including donepezil, galantamine, and rivastigmine, albeit leading to a slight improvement of symptoms, do not achieve a cure [2].

Aβ is one of the important pathological hallmarks of AD. A vast number of studies suggest that neurotoxicity by Aβ aggregation causes neurodegeneration in AD, leading to numerous lines of investigation focusing on removing Aβ or suppressing its production. Amyloid precursor protein (APP), which generates Aβ, is cleaved by a number of enzymes to produce different cleavage products. Under normal conditions, APP is cleaved by α-secretase, which results in the production of sAPPα and α-C-terminal fragment (αCTF); αCTF is subsequently cleaved by γ-secretase into APP-intracellular domain (AICD) and rapidly degraded to P83. Conversely, under pathological conditions, APP is first cleaved by β-secretase (BACE1) and not by α-secretase, to form sAPPβ and βCTF; this is followed by γ-secretase-mediated cleavage to form AICD and Aβ, which can aggregate easily. In other words, modulating the action of BACE1, a key Aβ-producing enzyme, is a potential therapeutic target that can reduce Aβ aggregation [3].

Cinnamon extract has been used in traditional medicine. Studies report that cinnamon extract exerts antioxidant [4], antidiabetic [5], and anti-inflammatory [6] effects in various diseases. Additionally, cinnamon extract was shown to exert an inhibitory effect on tau aggregation and Aβ oligomer formation, both associated with AD pathology [7]. Cinnamon is composed of various components such as trans-cinnamaldehyde (TCA), eugenol, and coumarin. Among them, TCA is present in levels as high as 72.7% compared to other volatile components [8]. TCA is a major active component of cinnamon that was shown to have antioxidant, cholesterol-lowering, antineoplastic, antibacterial, and antifungal properties. The potential therapeutic effects of TCA were also studied in several disease models of neurodegeneration, including 1-methyl-4-phenyl-1,2,3,6-tetrahydropyridine-induced Parkinson’s disease [9] and 6-hydroxydopamine-induced dopaminergic injury models [10]. However, no studies examined whether TCA, a single compound derived from cinnamon, might be an effective therapeutic in AD. Thus, we investigated the effect of TCA and related mechanisms against Aβ pathology in the five familial AD mutations (5XFAD) transgenic mouse.

## 2. Results

### 2.1. TCA Improves Cognitive Performance in 5XFAD Mouse Model of AD

**Figure 1:**
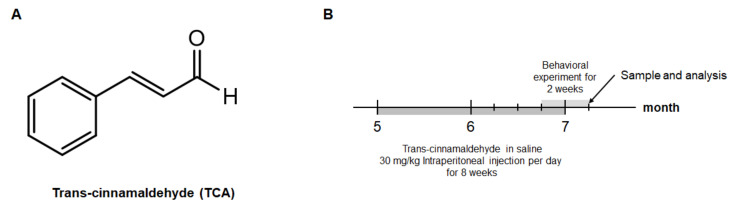
The chemical structure of trans-cinnamaldehyde (TCA) and experimental design. (**A**) TCA derived from the cinnamon contains an aldehyde structure. (**B**) Five-month-old 5XFAD was injected intraperitoneally with 30 mg/kg of TCA per day for eight weeks. After behavioral analyses, mice were sacrificed and brain samples were collected for further analysis.

**Figure 2:**
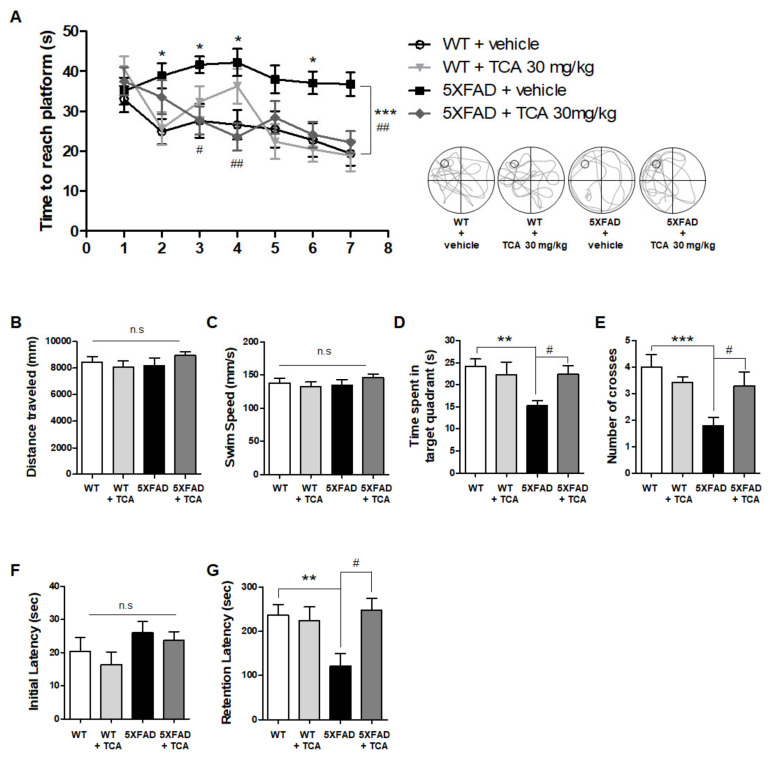
TCA improves cognitive performance in 5XFAD mice. (**A**) Spatial learning and memory were analyzed by estimating the time to reach a hidden platform for seven consecutive days. To analyze acquired memory, distance traveled (**B**), swim speed (**C**), time spent in the target quadrant (**D**), and number of target crosses (**E**) were identified by the probe test (removal of the platform). (**F**,**G**) Implicit memory was analyzed by measuring the time to enter the dark chamber. There was no difference in the initial latency (**F**), whereas there was a difference after the electric shock (**G**). All results are expressed as means ± standard error of the mean (S.E.M.) we used mice as follows: vehicle-treated wild-type (WT) group (*n* = 15), TCA-treated WT group (*n* = 8), vehicle-treated 5XFAD group (*n* = 15), TCA-treated 5XFAD group (*n* = 12). The data were analyzed by one-way analysis of variance with Tukey’s post hoc test. ** *p* < 0.01, *** *p* < 0.001, significantly different from the vehicle-treated WT group; # *p* < 0.05, ## *p* < 0.01, significantly different from the vehicle-treated 5XFAD group.

To examine whether TCA might improve cognitive performance in the 5XFAD mice, TCA-treated and vehicle-treated 5XFAD mice as well as the wild type (WT) littermates were assessed using Morris water maze (MWM) and passive avoidance test (PAT) (Figure 1). MWM was performed to identify spatial learning and memory, and the time to reach the hidden platform was determined. The vehicle-treated 5XFAD group consistently reached the platform later than the vehicle-treated WT group, starting from the first day. The TCA-treated 5XFAD group reached the platform faster than the vehicle-treated 5XFAD group on the third day and eventually found the platform more quickly than the vehicle-treated 5XFAD group on the seventh day (Figure 2A). On day eight, the probe task revealed that there was no difference in the swimming speed or the total distance traveled between the groups (Figure 2B,C). However, both the time spent in the target quadrant and the number of crossings across the platform were increased in the TCA-treated 5XFAD mice compared with the vehicle-treated 5XFAD mice (Figure 2D,E). PAT was performed to determine implicit memory, and the results revealed that there was no group-specific difference in the time to enter the dark chamber in the initial phase (Figure 2F). In the retention phase after the delivery of shock, the vehicle-treated 5XFAD group entered the dark chamber faster than the vehicle-treated WT group, whereas the TCA-treated 5XFAD group showed a significant increase in retention latency (Figure 2G). These results indicated that TCA improved cognitive impairment without a change in locomotor activity in 5XFAD mice.

### 2.2. TCA Reduces Aβ Deposition in the Brains of 5XFAD Mice

**Figure 3:**
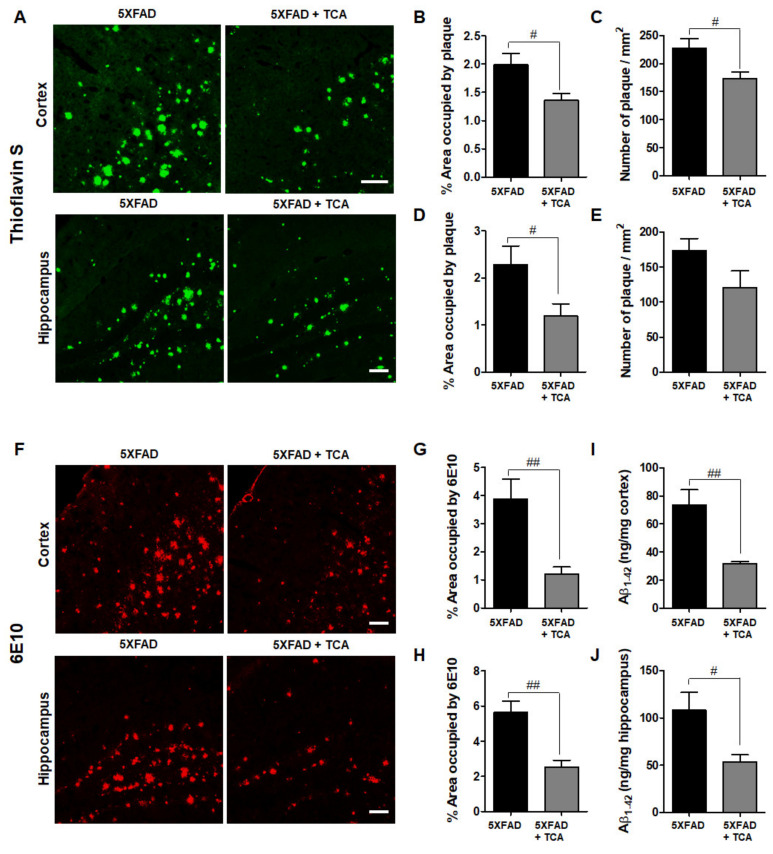
TCA reduces amyloid-β (Aβ) deposition in the brains of 5XFAD mice. (**A**) Representative images of thioflavin S staining. We used mice as follows: vehicle-treated 5XFAD mice (*n* = 6), TCA-treated 5XFAD mice (*n* = 5). Scale bar: 100 μm. (**B**,**D**) Areas occupied by Aβ plaques in the cortex (**B**) and hippocampus (**D**) of the 5XFAD mice treated with TCA or vehicle. (**C**,**E**) Number of plaques in the cortex (**C**) and hippocampus (**E**) of the 5XFAD mice treated with TCA or vehicle. (**F**) Representative images of Aβ staining with the 6E10 antibody, which specifically detects the Aβ 1–16 peptides. We used mice as follows: vehicle-treated 5XFAD mice (*n* = 6), TCA-treated 5XFAD mice (*n* = 5). Scale bar: 100 μm. (**G**,**H**) Quantification of 6E10-positive areas in cortex (**G**) and hippocampus (**H**) of the 5XFAD mice treated with TCA or vehicle. We used the same general area in different sections for the two different assays. (**I**,**J**) Enzyme-linked immunosorbent assay was performed to analyze Aβ 1–42 levels in the cortex (**I**) and hippocampus (**J**) of 5XFAD mice treated with TCA or vehicle. We used mice as follows: vehicle-treated 5XFAD mice (*n* = 7), TCA-treated 5XFAD mice (*n* = 5). Results are expressed as the mean ± S.E.M. Data were analyzed by Student’s *t* test. # *p* < 0.05, ## *p* < 0.01, significantly different from the vehicle-treated 5XFAD group.

Aβ deposition is a typical pathological finding in patients with AD. To examine the effect of TCA on Aβ deposition, brain tissue sections were stained with thioflavin S. The area of Aβ deposition as well as the number of Aβ plaques were significantly decreased in the TCA-treated 5XFAD mice compared with the vehicle-treated 5XFAD mice (Figure 3A–E). We also stained for Aβ deposition using 6E10 antibody, which specifically detects the Aβ 1–16 peptides, and obtained similar results with thioflavin S staining (Figure 3F–H). The enzyme-linked immunosorbent assay (ELISA) results also showed that the TCA administration could reduce the Aβ 1–42 levels in the brains of seven-month-old 5XFAD mice (Figure 3I,J). Together, these data suggested that TCA treatment could attenuate Aβ deposition in the brains of seven-month-old 5XFAD mice.

### 2.3. TCA Decreases BACE1 Levels in the Brains of 5XFAD Mice

**Figure 4:**
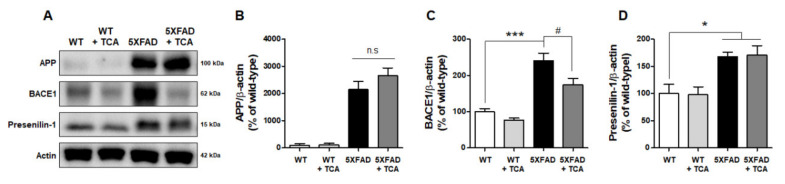
TCA decreases β-secretase (BACE1) levels in the brains of 5XFAD mice. (**A**) Representative immunoblotting for amyloid precursor protein (APP), BACE1, and presenilin1 (PS1) in brain tissues. (**B**–**D**) Quantification of APP (**B**), BACE1 (**C**), and PS1 (**D**) protein levels. We used mice as follows: vehicle-treated WT mice (*n* = 5), TCA-treated WT mice (*n* = 4), vehicle-treated 5XFAD mice (*n* = 5), TCA-treated 5XFAD mice (*n* = 4). The results are expressed as means ± S.E.M. Data were analyzed by one-way analysis of variance with Tukey’s post hoc test. * *p* < 0.05, *** *p* < 0.001, significantly different from the vehicle-treated WT group; # *p* < 0.05, significantly different from the vehicle-treated 5XFAD group.

We next aimed to determine the mechanism underlying TCA-mediated decrease of Aβ deposition in the brains of 5XFAD mice. First, we examined the protein expression of APP, presenilin-1 (PS1), and BACE1, which are associated with Aβ generation. As expected, Western blotting revealed that human APP was not expressed in WT mice but was detected in 5XFAD mice (Figure 4A). Conversely, BACE1 and PS1 were detected in both the WT and the 5XFAD mice. The comparison of the vehicle-treated 5XFAD and TCA-treated 5XFAD mice revealed no differences in APP and PS1 levels in the brain (Figure 4B,D). However, the expression of BACE1 was significantly increased in the 5XFAD mice compared to the age-matched WT mice. These levels were alleviated by TCA treatment, indicating that TCA could reduce BACE1 expression (Figure 4C, Supplementary Figure S1). Next, we assessed the mRNA levels of two Aβ-degrading enzymes, neprilysin (NEP), and insulin-degrading enzyme (IDE), to determine whether TCA involved Aβ degradation. The mRNA levels of NEP were decreased in the 5XFAD group compared with the WT group but did not recover after TCA administration (Supplementary Figure S2A). The mRNA levels of IDE did not differ between the WT and the 5XFAD groups but were slightly increased without statistical significance in the 5XFAD mice treated with TCA (Supplementary Figure S2B).

### 2.4. TCA Does Not Affect the Levels of Inflammatory Factors in the Brains of 5XFAD Mice

**Figure 5:**
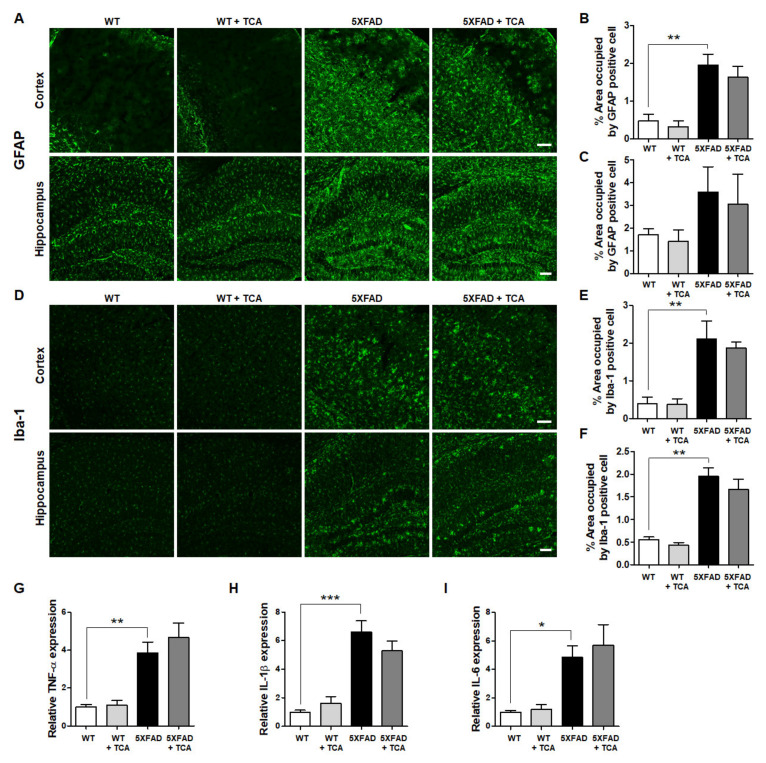
TCA does not affect inflammatory responses in the brains of 5XFAD. (**A**) Representative immunofluorescence images of glial fibrillary acidic protein (GFAP)-positive cells in the brains of 5XFAD mice. Scale bar: 100 μm. (**B**,**C**) Quantification of GFAP-positive areas in the cortex (**B**) and hippocampus (**C**) of the 5XFAD mice treated with TCA or vehicle. We used mice as follows: vehicle-treated WT mice (*n* = 3), TCA-treated WT mice (*n* = 4), vehicle-treated 5XFAD mice (*n* = 4), TCA-treated 5XFAD mice (*n* = 4). (**D**) Immunofluorescence images of ionized calcium-binding adapter molecule 1 (Iba-1)-positive cells in the brains of 5XFAD mice. Scale bar: 100 μm. (**E**,**F**) Quantification of Iba-1-positive areas in the cortex (**E**) and hippocampus (**F**) of the 5XFAD mice treated with TCA or vehicle. We used mice as follows: vehicle-treated WT mice (*n* = 3), TCA-treated WT mice (*n* = 4), vehicle-treated 5XFAD mice (*n* = 4), TCA-treated 5XFAD mice (*n* = 5). (**G**–**I**) qRT-PCR was performed to detect pro-inflammatory cytokines. Tumor necrosis factor-α (TNF-α) (**G**), interleukin-1β (IL-1β) (**H**), and IL-6 (**I**) levels were increased in the brain of 5XFAD mice compared to the WT mice, although there were no decreases in their levels in the TCA-treated group. We used mice as follows: vehicle-treated WT mice (*n* = 6), TCA-treated WT mice (*n* = 4), vehicle-treated 5XFAD mice (*n* = 6), TCA-treated 5XFAD mice (*n* = 5). Data are expressed as means ± S.E.M. and analyzed by one-way analysis of variance with Tukey’s post hoc test. * *p* < 0.05, ** *p* < 0.01, *** *p* < 0.001, significantly different from the vehicle-treated WT group.

BACE1 can be activated by pro-inflammatory cytokines released by astrocytes and microglia, such as tumor necrosis factor-α (TNF-α), interleukin-1β (IL-1β), and IL-6 [11]. To investigate whether TCA could reduce BACE1 levels via regulating neuroinflammation, we examined the extent of gliosis by assessing astrocytes and microglia. Mouse brain slices were stained with antibodies against anti-ionized calcium-binding adapter molecule 1 (Iba-1), a microglial marker, and glial fibrillary acidic protein (GFAP), an astrocytic marker. As expected, astrocytic and microglial activation was significantly higher in the 5XFAD mice than the age-matched WT mice. Although there was a slight decrease of GFAP- and Iba-1-positive cells in the TCA-treated 5XFAD group, this was not statistically significant, indicating that TCA treatment was not associated with a change in gliosis (Figure 5A–F). We also confirmed these results by assessing the gene expression levels of the pro-inflammatory cytokines including TNF-α, IL-1β, and IL-6 (Figure 5G–I). These results suggested that TCA did not impact neuroinflammation in the 5XFAD mice and that the reduced BACE1 levels in the TCA-treated 5XFAD mice were not related to neuroinflammation.

### 2.5. TCA Activates the SIRT1-PGC1α-PPARγ Pathway That Regulates the Expression of BACE1

**Figure 6:**
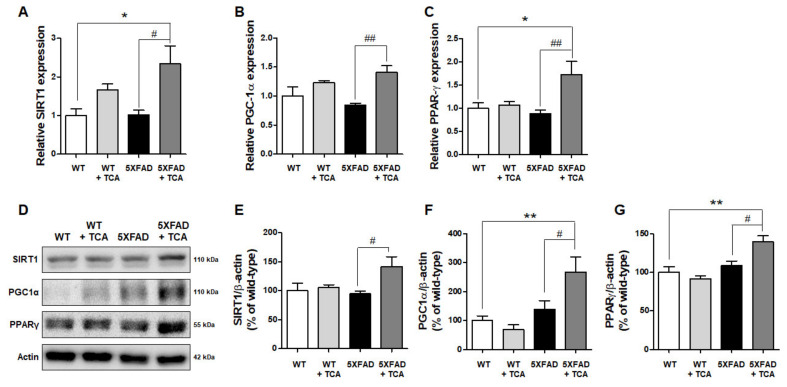
TCA activates the silent information regulator 1 (SIRT1)-peroxisome proliferator-activated receptor γ (PPARγ) coactivator 1α (PGC1α)-PPARγ pathway that regulates the expression of BACE1 in the brains of 5XFAD mice. (**A**–**C**) Analysis of mRNA expression levels of SIRT1, PGC1α, and PPARγ by qRT-PCR. We used mice as follows: vehicle-treated WT mice (*n* = 6), TCA-treated WT mice (*n* = 4), vehicle-treated 5XFAD mice (*n* = 6), TCA-treated 5XFAD mice (*n* = 5). SIRT1 (**A**) and PPARγ (**C**) levels were significantly increased in the TCA-treated 5XFAD group compared to the WT and vehicle-treated 5XFAD groups. (**B**) PGC1α was decreased in the 5XFAD group compared to the WT group and slightly recovered in the TCA-treated group. (**D**) Representative immunoblotting results to determine the protein levels of SIRT1, PGC1α, and PPARγ. (**E**–**G**) Quantification of SIRT1 (**E**), PGC1α (**F**), and PPARγ (**G**) in the brains of mice. We used mice as follows: vehicle-treated WT mice (*n* = 5), TCA-treated WT mice (*n* = 4), vehicle-treated 5XFAD mice (*n* = 5), TCA-treated 5XFAD mice (*n* = 4). Data were expressed as means ± S.E.M. and analyzed by one-way analysis of variance with Tukey’s post hoc test. * *p* < 0.05, ** *p* < 0.01, significantly different from the vehicle-treated WT group; # *p* < 0.05, ## *p* < 0.01, significantly different from the vehicle-treated 5XFAD group.

Another possible mechanism underlying BACE1 downregulation by TCA might involve signaling pathways. The silent information regulator 1 (SIRT1)-peroxisome proliferator-activated receptor γ (PPARγ) coactivator 1α (PGC1α)-PPARγ pathway is known to regulate the expression of BACE1 [12]. To examine whether TCA might be exerting its effect on BACE1 levels via this signaling pathway, we determined the mRNA levels of SIRT1, PGC1α, and PPARγ. We found that the mRNA levels of SIRT1 and PPARγ were significantly increased in the TCA-treated 5XFAD group than the vehicle-treated 5XFAD group, although their levels were not lower in the vehicle-treated 5XFAD group compared with the vehicle-treated WT group (Figure 6A,C). PGC1α mRNA levels tended to decrease slightly in the brains of the 5XFAD mice and exhibited recovery in the TCA-treated 5XFAD mice (Figure 6B). To confirm whether the changes in gene expression could lead to alterations in protein levels, we performed Western blot analysis and found a significant increase in protein levels of SIRT1, PGC1α, and PPARγ in the TCA-treated 5XFAD group compared with the vehicle-treated 5XFAD group (Figure 6D–G). 

## 3. Discussion

The main objective of the present study was to evaluate the effect of TCA, a major active compound of cinnamon [6] and to examine its mechanism of action in improving AD-related pathology. We found that TCA activated the SIRT1-PGC1α-PPARγ pathway, resulting in decreased BACE1 levels and attenuated symptoms associated with AD without any effects on the WT mice. To the best of our knowledge, this is the first study to demonstrate that TCA ameliorated Aβ-associated pathology through BACE1 regulation in a mouse model of AD.

In this study, we evaluated the therapeutic effects of TCA in the 5XFAD mice and demonstrated that the 5XFAD mice receiving TCA showed improvement in cognitive ability and decreases of Aβ deposition. Frydman-Marom et al. reported that aqueous cinnamon extract was an effective inhibitor of toxic Aβ oligomer formation [7]. The authors also showed that cinnamon extract inhibited Aβ aggregation in the hippocampus of an AD mouse model [7]. However, the study did not determine the specific component of the cinnamon extract involved in the reduction of Aβ aggregation or the underlying mechanisms. In the current study, we confirmed that TCA, a major active compound in cinnamon, could inhibit Aβ deposition in the brain of AD mouse model. To determine whether TCA directly alleviates Aβ aggregation, we first performed thioflavin T assay but did not observe an anti-aggregation effect of TCA (data not shown). Therefore, we hypothesized that the TCA-mediated reduction of Aβ in our AD model might be related to the generation of Aβ.

The first step in Aβ generation is APP cleavage by BACE1, an aspartyl protease bound to the plasma membrane by a characteristic domain near the C-terminal [13]. A previous study showed that BACE1 was elevated in the brains of AD patients and that the activity of BACE1 was correlated with Aβ plaques [14]. These findings suggest that BACE1 might have a major impact on accelerating Aβ deposition. Aβ production was also increased in association with the elevation of BACE1 following traumatic brain injury and hypoxia [15,16]. BACE1-null mice engineered to overexpress human APP exhibited significant improvements in cognitive and behavioral deficits and dramatically reduced Aβ 1–40 and Aβ 1–42 levels [17]. Thus, BACE1 is considered as a therapeutic target for modulating the production of Aβ and improving AD symptoms. In this study, we found that TCA treatment reduced Aβ deposition via the inhibition of BACE1 expression, resulting in improved cognitive performance in 5XFAD mice.

Expression of BACE1 is regulated by a variety of factors including inflammation, hypoxia, and endoplasmic reticulum stress [18]. Numerous studies examined the factors that positively or negatively regulate the gene expression of BACE1 by acting on its promoter [19,20]. The BACE1 promoter domains act as binding sites for many transcription factors including cAMP response element binding protein [21], nuclear factor kappa-light-chain-enhancer of activated B cells [22], hypoxia-inducible factor 1 [16], and PPARγ [23]. These transcription factors affect the transcriptional activity of BACE1. In our experiments, TCA had a slight effect on inflammation. As shown in Figure 5, activation of astrocyte and microglia and expression of IL-1β mRNA slightly decreased by TCA-treatment in 5XFAD mice. However, it did not reach a statistical significance under our experimental conditions. Interestingly, the current study demonstrated that the reduced BACE1 levels by TCA in the 5XFAD mice were associated with the increased expression of PPARγ, one of the regulators of BACE1. We analyzed the signaling pathway, which is known to regulate BACE1 transcription, to further elucidate the underlying mechanism. It was discovered by Juan-E Li et al. that TCA can induce activation of PPARγ and retinoid X receptor (RXR) [24]. After heterodimerization with RXR, PPARγ binds to a specific promoter region of the target gene to peroxisome proliferator response elements (PPREs) and acts as transcription factor [25]. Heneka et al. showed that acute administration of a PPARγ agonist reduced both the mRNA and protein levels of BACE1 [26]. Katsouri et al. also showed that PGC1α, the coactivator of PPARγ, regulated BACE1 expression in a PPARγ-dependent manner [27]. In another study, it was demonstrated that SIRT1 was directly involved in the activation of PGC1α [28]. Qiang et al. showed that PPARγ is activated directly by the deacetylation of SIRT1 [29]. Finally, Ruishan et al. reported that these three related factors formed a complex with each other, interacting with the PPRE-RXR site at the promotor of BACE1 gene, acting as a negative regulator [12]. Similar to what was observed in previous studies, we found that TCA reduces Aβ deposition by downregulating BACE1, and it might be related to the activation of SIRT1-PGC1α-PPARγ pathway in 5XFAD mice. However, further studies are needed to determine whether TCA directly promotes their transcription or induces their expression level through other pathways related to the transcription of these three negative regulators of BACE1.

## 4. Materials and Methods

### 4.1. Materials

TCA, thioflavin S, triton X-100, paraformaldehyde, phosphate buffer, phosphate-buffered saline (PBS, pH 7.4), and tris-buffered saline (TBS) were purchased from Sigma Aldrich (St. Louis, MO, USA). RIPA buffer and protease/phosphatase inhibitor cocktail were obtained from Thermo Fisher Scientific (Waltham, MA, USA). Fluorescence mounting medium was purchased from Dako (Santa Clara, CA, USA). The antibodies used in the experiments are listed in the Supplementary Table S1.

### 4.2. Animals and Treatment

5XFAD mice were purchased from Jackson Laboratory (stock number: 034840-JAX, Bar Harbor, ME, USA). Mice were housed in plastic containers under constant temperature (23 ± 1 °C) and humidity (60 ± 10%), in a 12-h light/dark cycle with free access to food and water. Five-month-old 5XFAD female mice (22 ± 2 g) and male mice (30 ± 2 g) were injected intraperitoneally with 30 mg/kg of TCA in saline (0.9% NaCl) per day for eight weeks. It was also applied to WT female and male mice of the same age. In each group, one third was female and the rest were male. All animal studies were performed in accordance with the “Principles of Laboratory Animal Care” (National Institutes of Health publication number 80–23, revised 1996) and approved in 2018 by the Animal Care and Use Guidelines Committee of Kyung Hee University (approval number: KHUASP(SE)-17-126-1).

### 4.3. Behavioral Studies

We performed behavioral studies to assess spatial learning and memory in the MWM as previously described [30]. Animals were given three trials per day for 7 days to learn the task. At 8 days, animals were given a probe trial in which the platform was removed. Each mouse was placed into one quadrant of the pool and allowed to swim for 60 s. All trials were recorded using a charge-coupled device camera connected to a video monitor and a computer.

PAT consists of two boxes of the same environment in which an animal receives an electric shock when it spontaneously moves from a compartment lit with a light bulb to a dark compartment. This one-trial learning procedure typically lasts less than 30 s. Twenty-four hours after the learning phase, the animals were placed back in the brightly lit compartment for the retest phase, and latency in stepping into the darkened compartment was measured. The maximum possible latency was arbitrarily set at 300 s.

### 4.4. Brain Tissue Preparation

Mice were euthanized after behavioral testing by administration of a mixture of ketamine and xylazine in 0.9% NaCl as the anesthetic, and cardiac perfusion was performed immediately using 4% paraformaldehyde in PBS. After perfusion, brains were removed, post-fixed overnight at 4 °C, and incubated in 30% sucrose at 4 °C until equilibration. Sequential 30-μm-thick coronal sections were prepared by a cryostat (CM1850; Leica, Wetzlar, Germany) and stored at 4 °C.

### 4.5. Thioflavin S Staining

Free-floating sections were incubated for 10 min in 1% thioflavin S dissolved in 50% ethanol, followed by two washes with 50% ethanol for 5 min each and one wash with distilled water for 5 min; the sections were then mounted using mounting medium.

### 4.6. Immunofluorescence

Immunofluorescence was done according to previously described procedures [31]. The following antibodies were used: anti-6E10, anti-Iba-1, anti-GFAP. For visualization, the sections were incubated with Alexa Fluor 488- or Alexa Fluor 594- conjugated secondary antibodies for 1 h at room temperature. Images of the sections were captured using laser-scanning confocal microscopy (K1-Fluo; Nanoscope Systems, Daejeon, Korea) or a BX51 immunofluorescence microscope (Olympus, Tokyo, Japan). 

### 4.7. Aβ 1–42 ELISA

Aβ 1–42 ELISA was performed using fluorescent-based kit (Invitrogen, Camarillo, CA, USA) and appropriate Aβ standards according to the manufacturer’s protocol. In each mouse, the left half of the cortex or hippocampus was homogenized in buffer containing 50 mM tris and 5 M guanidine HCl (pH 8.0). Homogenates were mixed at room temperature for 4 h and diluted in PBS containing 5% BSA, 0.03% Tween 20, and protease inhibitor cocktail.

### 4.8. RNA Isolation and Quantitative Real-Time Polymerase Chain Reaction (qRT-PCR)

qRT-PCR was performed to measure mRNA transcripts of cytokines. Briefly, total RNA was extracted from the left half of the cortex or hippocampus using the Hybrid-R™ (GeneAll, Seoul, Korea), and RNA concentrations were measured using a Nanodrop ND-1000 spectrophotometer (Thermo Fisher Scientific). RNA samples (3 μg) were converted to cDNA using TOPscript™ RT DryMIX (Enzynomics, Daejeon, Korea). cDNA was amplified and quantified by qRT-PCR using TOPreal™ qPCR 2X PreMIX (SYBR Green; Enzynomics) and the CFX Connect real-time PCR system (Bio-Rad Laboratories, Hercules, CA, USA). Primers are described in Supplementary Table S2.

### 4.9. Western Blot Analysis

The right parts of brain tissue were extracted in RIPA buffer plus protease/phosphatase inhibitors. Samples were immunoblotted as previously described [32]. Protein detection was carried out using an ECL reagent (Bio-Rad Laboratories) and visualized by ChemiDoc (Bio-Rad Laboratories). For quantification of relative protein expression, the optical density of the protein band of interest was normalized to the optical density of β-actin detected on the same membrane.

### 4.10. Statistical Analysis

The results from three independent experiments were summarized and presented as means ± standard error of the mean (S.E.M.). Statistical comparisons between two groups were performed with Student’s *t* test. In cases where more than two groups were compared with each other, a one-way analysis of variance followed by Tukey’s multiple comparison post hoc test, and *p*-values less than 0.05 were considered to indicate statistically significant differences from the controls, as indicated by an asterisk. The GraphPad Prism 5.0 software (Graph Pad Software, San Diego, CA, USA) was used for all statistical analyses. No significant difference between male and female mice in all analyses; thus, data obtained from male and female mice were combined.

## 5. Conclusions

The present study reveals a therapeutic effect of TCA in an animal model of AD. We showed that TCA inhibited Aβ deposition by modulating the expression of BACE1 in the 5XFAD mice, thereby inhibiting cognitive decline. Furthermore, the modulating effect of TCA on BACE1 levels was associated with the upregulation of PPARγ, SIRT1, and PGC1α, negative regulators of BACE1 that ultimately led to the improvement in cognitive impairment without any effects in the WT mice. In the future, molecular study as well as prolonged clinical trials are required to establish the therapeutic safety and efficacy of TCA. Taken together, our findings strongly suggest the possibility that TCA might be an effective candidate for the treatment of AD by reducing Aβ deposition, a main pathological hallmark of AD.

## Supplementary Materials

Supplementary Materials can be found at https://www.mdpi.com/1422-0067/21/12/4492/s1. Figure S1: mRNA expression of BACE1; Figure S2: mRNA expression of Aβ-degrading enzymes; Table S1: Information of immunostaining antibodies used in this study; Table S2: Information of qRT-PCR primers used in this study.

## Abbreviations

AD	Alzheimer’s disease
Aβ	amyloid-β
BACE1	β-secretase
APP	amyloid precursor protein
TCA	trans-cinnamaldehyde
SIRT1	silent information regulator 1
PGC1α	peroxisome proliferator-activated receptor γ coactivator 1α
PPARγ	peroxisome proliferator-activated receptor γ
PS1	presenilin-1
TNF-α	tumor necrosis factor-α
IL-1β	interleukin-1β
IL-6	interleukin-6
Iba-1	ionized calcium-binding adapter molecule 1
GFAP	glial fibrillary acidic protein
RXR	retinoid X receptor
PPRE	peroxisome proliferator response elements
