# Supplementary material for: Trans-Cinnamaldehyde Alleviates Amyloid-Beta Pathogenesis via the SIRT1-PGC1α-PPARγ Pathway in 5XFAD Transgenic Mice
Source: Int J Mol Sci. 2020 Jun 24;21(12):4492. doi: 10.3390/ijms21124492 (PMC7352815; doi:10.3390/ijms21124492)
Supplement: Supplementary file 1 [file ijms-21-04492-s001.pdf]

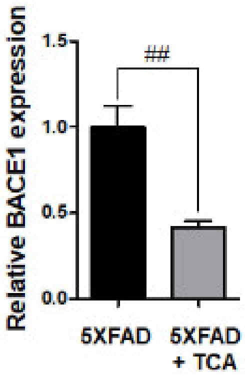

Supplementary Figure S1. mRNA expression of β-secretase (BACE1). We used mice as follows: vehicle-treated 5XFAD mice (n = 4), TCA-treated 5XFAD mice (n = 4). Results are expressed as the mean ± S.E.M. Data were analyzed by Student's t test. ## $p < 0.01$, significantly different from the vehicle-treated 5XFAD group.

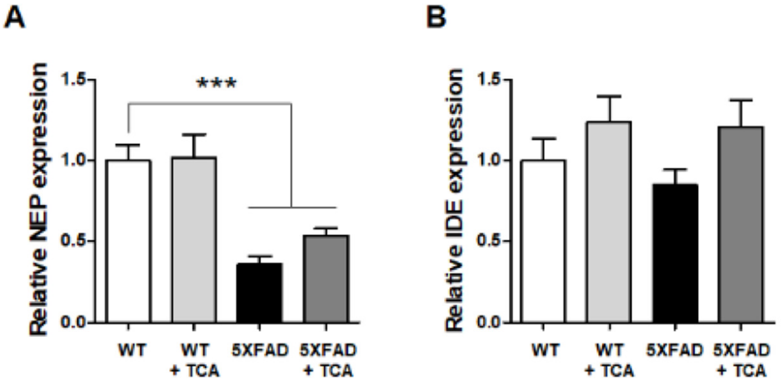

Supplementary Figure S2. mRNA expression of Aβ-degrading enzymes. mRNA levels of neprilysin (**A**) and insulin degrading enzyme (IDE) (**B**) were analyzed by quantitative real-time polymerase chain reaction. We used mice as follows: vehicle-treated WT mice (n = 6), TCA-treated WT mice (n = 4), vehicle-treated 5XFAD mice (n = 6), TCA-treated 5XFAD mice (n = 5). The results are expressed as means ± S.E.M. Data were analyzed by one-way analysis of variance with Tukey's post hoc test. ***p < 0.001, significantly different from the WT group.

Supplementary Table S1. Information of immunostaining antibodies used in this study.

Antibody	Host	Source	Catalog No.	RRID	Application
6E10	Mouse	Biolegend, San Diego, CA, USA	803001	AB_2564653	WB, 1:1000 IHC, 1:500
BACE1	Mouse	Millipore, Burlington, MA, USA	MAB5308	AB_95207	WB, 1:1000
PS1	Rabbit	Cell signaling, Danvers, MA, USA	5643s	AB_10706356	WB, 1:1000
Iba-1	Rabbit	Wako Chemical, Richmond, VA, USA	019-19741	AB_839504	IHC, 1:500
GFAP	Rabbit	Dako, Santa Clara, CA, USA	Z0334	AB_10013382	IHC, 1:500
SIRT1	Rabbit	Cell signaling, Danvers, MA, USA	2028s	AB_1196631	WB, 1:1000
PGC1α	Mouse	Santa Cruz Biotechnology, Dallas, TX, USA	sc-518025	-	WB, 1:1000
PPARγ	Rabbit	Cell signaling, Danvers, MA, USA	2443s	AB_823598	WB, 1:1000
β-actin	-	Santa Cruz Biotechnology, Dallas, TX, USA	sc-47778 HRP	AB_2714189	WB, 1:5000
Mouse IgG	Goat	Santa Cruz Biotechnology, Dallas, TX, USA	sc-2005	AB_631736	WB: 1:5000
Rabbit IgG	Goat	Santa Cruz Biotechnology, Dallas, TX, USA	sc-2054	AB_631748	WB: 1:5000
Alexa Fluor 488	Goat	Thermo Fisher Scientific, Waltham, MA, USA	A11001	AB_2534069	IHC, 1:1000

Supplementary Table S2. Information of qRT-PCR primers used in this study.

Gene	Primers	
NEP	Forward	5′-GAA ATT CAG CCA AAG CAA GC-3′
	Reverse	5′-GAT TTC GGC CTG AGG AAT AA-3′
IDE	Forward	5′-CCA AGA AGG CAT CGA CGT AA-3′
	Reverse	5′-GAT GCT CTT CCT GGA AAG GG-3′
TNFα	Forward	5′-GAT TAT GGC TCA GGG TCC AA-3′
	Reverse	5′-GCT CCA GTG AAT TCG GAA AG-3′
IL-1β	Forward	5′-CCC AAG CAA TAC CCA AAG AA-3′
	Reverse	5′-GCT TGT GCTCTG CTT GTG AG-3′
IL-6	Forward	5′-CCG GAG AGG AGA CTT CAC AG-3′
	Reverse	5′-TTG CCA TTG CAC AAC TCT TT-3′
SIRT1	Forward	5′-ACG CTG TGG CAG ATT GTT ATT A-3
	Reverse	5′-TTG AAG AAT GGT CTT GGG TCT T-3′
PGC1α	Forward	5′-AAT GAA TGC AGC GGT CTT AG-3′
	Reverse	5′-GTC TTT GTG GCT TTT GCT GT-3′
PPARγ	Forward	5′-TGT GGG GAT AAA GCA TCA GGC-3′
	Reverse	5′-CCG GCA GTT AAG ATC ACA CCT AT-3′
